# The Effect of Dietary Lycopene Supplementation on Drip Loss during Storage of Lamb Meat by iTRAQ Analysis

**DOI:** 10.3390/antiox10020198

**Published:** 2021-01-29

**Authors:** Bo Wang, Chen-chen Xu, Ce Liu, Yang-hua Qu, Hao Zhang, Hai-ling Luo

**Affiliations:** 1 State Key Laboratory of Animal Nutrition, College of Animal Science and Technology, China Agricultural University, NO.2 Yuanmingyuan West Road, Haidian, Beijing 100193, China; wangboforehead@163.com (B.W.); chenxu30510@cau.edu.cn (C.-c.X.); liuceshiyan@163.com (C.L.); quyanghua@cau.edu.cn (Y.-h.Q.); 2 Beijing Advanced Innovation Center for Food Nutrition and Human Health, College of Food Science and Nutritional Engineering, China Agricultural University, No.17 Qinghua East Road, Haidian, Beijing 100083, China; zhanghaocau@cau.edu.cn

**Keywords:** lycopene, drip loss, lipid and protein oxidation, iTRAQ, storage period, lamb meat

## Abstract

This study was designed to investigate the impact of dietary lycopene (antioxidant extracted from tomato) supplementation on postmortem antioxidant capacity, drip loss and protein expression profiles of lamb meat during storage. Thirty male Hu lambs were randomly divided into three treatment groups and housed in individual pens and received 0, 200 or 400 mg·kg^−1^ lycopene in their diet, respectively. All lambs were slaughtered after 3 months of fattening, and the longissimus thoracis (LT) muscle was collected for analyses. The results indicated that drip loss of LT muscle increased with storage days (*P* < 0.05). After storage for 7 days, significantly lower drip loss of meat was found in fed the lycopene-supplemented diet (*P* < 0.05). Dietary lycopene supplementation increased the activity of antioxidant enzymes (total antioxidant capacity (T-AOC), superoxide dismutase (SOD), glutathione peroxidase (GSH-Px), catalase (CAT)) (*P* < 0.05) and decreased the thiobarbituric acid reactive substance (TBARS) and carbonyl contents (*P* < 0.05). During the storage period (days 0, 5 and 7), a number of differentially abundant proteins (DAPs), including oxidases, metabolic enzymes, calcium channels and structural proteins, were identified based on iTRAQ data, with roles predominantly in carbon metabolism, oxidative phosphorylation, cardiac muscle contraction and proteasome pathways, and which contribute to decreased drip loss of lamb meat during storage. It can be concluded that dietary lycopene supplementation increased antioxidant capacity after slaughter, and the decreased drip loss during postmortem storage might occur by changing the expression of proteins related to enzyme activity and cellular structure in lamb muscle.

## 1. Introduction

Oxidation reactions not only have important functions in living cells, but they also occur in postmortem changes to muscle [1]. The safety, nutritional value and shelf life of meat can be jeopardized by oxidation reactions [2,3]. Previous studies have reported that both lipid and protein oxidative processes appeared to decrease water-holding capacity (WHC) [4,5] by affecting structural and compositional changes of membranes and modulating the fragmentation and aggregation of proteins in meat [6,7]. Drip loss, as an indicator of the meat WHC, is one of the important parameters for both the meat industry and the consumer to evaluate meat quality. For the meat industry, drip loss of meat is known to influence its technological quality (such as processing yield) and economic benefits [8]. For the consumer, higher drip loss reduces the tenderness, juiciness and sensory quality of the meat, causing lower consumer acceptance [9,10,11].

Antioxidants act as protective agents by quenching reactive oxygen species to protect the organelle/cell/tissue from oxidative damage [12]. Recently, considerable attention has been focused on improving meat quality through inhibiting or reducing oxidative deterioration by dietary antioxidant supplementation, especially natural antioxidants extracted from plants because of their efficiency and safety to humans [13,14]. Moreover, consumers are concerned about the potential risk to health by using synthetic antioxidants in meat or animals’ diet [15], so they prefer to consume meat products with natural ingredients [16]. Thus, antioxidants from plant extracts are more in line with consumers’ preferences.

It was reported that lycopene might be one of the most potent antioxidants among dietary carotenoids, and its singlet-oxygen-quenching rate was twice that for ß-carotene and 100 times of that for α-tocopherol in vitro chemical trail [17]. Østerlie and Lerfall (2005) also reported that lycopene from tomato products added to minced beef increased the meat storage stability and showed a better color and health benefits [18]. Our previous studies indicated that dietary lycopene supplementation was helpful to produce meat with less fat and higher PUFA content and antioxidative capacity of fresh meat in Bamei sheep [19,20]. Recently, we reported that dietary lycopene supplementation prevented lipids and proteins from oxidation, enhanced color stability, increased antioxidants (vitamin A, VA; and vitamin E, VE) accumulation in Hu lambs’ meat during storage [21]. Other research also reported that drip loss showed a positive relationship with time in storage [22,23]. However, whether the possible antioxidant capacity of lycopene supplemented in the diet might help to control drip loss during the storage of meat still unknown.

Moreover, in recent years, new bioinformatics analysis technologies were applied to discover candidate bioinformation of drip loss involved and provide potential biomarkers for meat quality evaluation. A large number of drip loss-related quantitative trait loci have been identified using the linkage mapping method and genome-wide association studies (GWASs) [24,25]; many candidate genes and some potential microRNAs which related to drip loss also have been reported using RNA sequencing [26,27]. However, the key regulatory proteins involved in drip loss during storage remain unclear.

Therefore, we hypothesized that dietary lycopene supplementation might decrease drip loss by increasing the antioxidant ability of lamb meat after slaughtering and regulating differentially abundant proteins (DAPs) of lamb meat during storage. The aim of the present study was to examine the effect of dietary lycopene supplementation on postmortem antioxidant capacity, drip loss of lamb meat and key DAPs related to drip loss during storage to test the above hypothesis.

## 2. Materials and Methods

### 2.1. Experimental Design and Treatments

The experiment was performed with a completely randomized design. All experimental animals were randomly divided into three groups and each group was fed one of the following diets: control diet (CON) or 200 (LP200) or 400 mg·kg^−1^ (LP400) lycopene supplementation based on the dry matter of the control diet. The levels of supplementary lycopene in the diet were based on the results of a previous study conducted by our team [19,20]. The lycopene powder (a commercial product) used in this experiment was purchased from Shanxi Sciphar Biotechnology Co. Ltd. (Xi’an, China). The chemical composition of the purchased lycopene product contained 3.1% moisture, 10.84% total lycopene, 13.2% protein, 50.67% carbohydrate, 14.6% fiber, 2.7% fat and 4.2% ash by analysis. Other antioxidant ingredients such as vitamin A, vitamin E, vitamin C, Se, Zn, Cu and Mn were not detected (these ingredients were checked for before conducting the experiment to ensure that no other antioxidants in the lycopene powder affect the results). In addition, the total lycopene was further analyzed to contain 75.5% all-E-lycopene, 19.0% 5Z-lycopene and 5.5% other related compounds.

### 2.2. Animals and Diets

Thirty healthy 3-month-old male Hu lambs with similar body weights (22 ± 0.5 kg) were selected and divided into 3 groups to conduct the trial. Each group had 10 lambs. All lambs were housed in individual pens (pen size: 3.0 × 1.0 × 1.5 m) with continuous access to water. The lycopene powder was mixed with the concentrate and thus fed to the animals to make sure that the lambs consumed the entire amount of lycopene prescribed to them by the experimental design. Roughage was provided after the concentrate feed finished. The concentrate-to-roughage ratio was 50:50. The experimental period started after 10 days of adaptation to the diets and lasted 3 months for fattening. After fattening, the final body weights for the CON, LP200 and LP400 groups were 40.94 ± 1.00, 43.42 ± 0.58 and 43.36 ± 0.81 kg (*P* = 0.065), respectively. Average daily dry matter intake, average daily gain and feed conversion ratio for CON, LP200 and LP400 were 1.17 ± 0.01, 1.17 ± 0.02 and 1.19 ± 0.01 kg (*P* = 0.593), 167.45 ± 9.75, 190.09 ± 5.13 and 189.50 ± 6.27 g (*P* = 0.061), 7.15 ± 0.32, 6.19 ± 0.10 and 6.34 ± 0.20 (*P* = 0.011), respectively [28]. 

The ingredients of the control diet were formulated according to NRC requirements [29] (Table 1). Chemical compositions including crude protein, ether extract, calcium and phosphorus were measured using Association of Official Analytical Chemists (AOAC) methods [30]. Neutral detergent fiber (NDF) and acid detergent fiber (ADF) contents were determined as described by Van Soest et al. (1991) [31]. High-performance liquid chromatography (HPLC, Agilent 1200 series) and atomic fluorescence spectrophotometry were used separately to measure VA and VE [20] and Se and VC contents in the lycopene powder [32,33]. The contents of Zn, Cu and Mn in the lycopene powder were measured using an atomic absorption spectrometer (PE 5100 Z, Germany).

### 2.3. Sample Collection

All lambs were fasted for 12 h and then euthanized by exsanguination following electrical stunning; the euthanasia and the subsequent evisceration procedures were according to the commercial standard (DB11-T 399-2006). The pre-slaughter live weight of CON, LP200 and LP400 lambs was 39.59 ± 0.96, 42.07 ± 0.64 and 42.04 ±0.80 kg (*P* = 0.062), respectively; the carcass weight was 20.69 ± 0.66, 21.97 ± 0.36 and 21.86 ± 0.49 kg (*P* = 0.172), respectively [28]. The carcasses were hung in a cold room at 2–3 °C for 1 h. After cooling, the longissimus thoracis (LT) muscle between the 6th and 13th ribs was dissected from the right side of each carcass and cut into 2 parts. One part was placed immediately into a −80 °C freezer and stored for meat chemical composition and antioxidant parameters determination on day 0; the other part was wrapped completely with a single layer of polyvinyl chloride film, placed on a polyethylene tray at 4 °C for 7 days and then stored at −80 °C until meat chemical composition was measured again (day 7). LT muscles at the same position on the left side were collected and cut into 2 parts for drip loss and iTRAQ analyses. One part was stored at 4 °C for determining drip loss on days 1, 3, 5 and 7, and the other part was prepared for iTRAQ analysis. The muscle sample for iTRAQ analysis was divided again into 3 parts and stored at 4 °C for 0, 5 and 7 days, respectively, and then transferred to a freezer at −80 °C until the analysis. 

### 2.4. Meat Chemical Composition Measurement

For meat chemical composition analysis (including moisture, protein, ether extract and ash), approximately 50 g of LT meat (m_1_) from each sample was minced, and the sample was wrapped with fresh-keeping film and pierced air holes; then, the samples were frozen at −20 °C overnight (more than 10 h). After precooling, samples were freeze-dried by means of a freeze dryer (FreeZone 6, Labconco, Kansas City, MO, USA) at −70 °C for 72 h, and the weight of the freeze-dried samples was recorded (m_2_). Then, moisture content was calculated according to the following formula: moisture (%) = (m_1_ − m_2_)/m_1_ × 100. Crude protein and fat content were determined using the Automatic Kjeldahl Apparatus (Kjeltec TM 2300, Foss, Hilleroed, Denmark) and the Automatic Extraction Instrument (XT15, Ankom, New York City, NY, USA) as described by AOAC [24], respectively.

### 2.5. Drip Loss Analysis

Drip loss was estimated as described by Honikel (1998) [34]. Approximately 80 g (W_1_) of each LT sample was packaged in a transparent and inflated plastic bag with no contact between the sample and the bag. The packaged samples were then suspended in a 4 °C refrigerator. After hanging for 1, 3, 5 and 7 d, respectively, the samples were taken out of the bag, wiped gently to remove the surface water and weighed (W_n_). Drip loss was expressed as a percentage of the initial weight, according to the following formula: drip loss (%) = (W_1_ − W_n_)/W_1_ × 100.

### 2.6. Antioxidant Indices Determination

Total antioxidant capacity (T-AOC): The T-AOC was measured based on the fact that the antioxidant system could reduce Fe^3+^ to Fe^2+^, which can bind with the phenanthroline complex that could be detected spectrophotometrically at 520 nm, as described by Lee et al. (1981) [35]. One unit of T-AOC was defined as a 0.01 increase in optical density by one gram of protein sample per minute at 37 °C.

Superoxide dismutase (SOD): SOD activity was determined using a commercial kit (Nanjing Jiancheng Biotechnology, Co., Ltd., Jiangsu, China) based on the utilization of tetrazolium salt for the detection of superoxide radicals generated by xanthine oxidase. One unit of SOD activity was defined as the activity that inhibits 50% dismutation of the superoxide radical. SOD activity was expressed as units of SOD per mg of protein.

Glutathione peroxidase (GSH-Px): GSH-Px activity was measured according to the fact that GSH-Px was able to promote the reaction of H_2_O_2_ with GSH to produce H_2_O and oxidized glutathione. One unit of GSH-Px activity can be expressed by its enzymatic reaction rate and calculated by the consumption of 1 μmol/L GSH in one gram of protein per minute.

Catalase (CAT): The activity of CAT was measured as a decrease in hydrogen peroxide at 240 nm for 5 min as per the Aebi method [36]. One unit of CAT activity was defined as the amount of CAT needed to decompose 1 mmol H_2_O_2_ per minute.

### 2.7. Lipid and Protein Oxidation Indices

Thiobarbituric acid reactive substance (TBARS), carbonyl and sulfhydryl contents were determined as described by Xu et al. (2019) [21]. For calculating the TBARS and carbonyl concentrations, absorbance was measured at 532 and 370 nm, respectively, using a 752-type spectrophotometer (Spectral Instrument Co. Ltd., Shanghai, China). The amount of TBARS was presented as mg malondialdehyde kg^−1^ meat, while that of carbonyl was shown as nmol mg^−1^ protein. The Multiskan Mk3 Microplate reader (Thermo Fisher Scientific Co., Beverly, MA, USA) was used to measure absorbance at 405 nm for the evaluation of the sulfhydryl content. The sulfhydryl levels presented were indicated as nmol total sulfhydryls mg^−1^ protein.

### 2.8. iTRAQ Analysis

The iTRAQ method was used to compare differences in the proteomic profiles of the LT muscle samples collected from animals with or without dietary lycopene supplementation.

#### 2.8.1. Protein Extraction, Quantification, Digestion and iTRAQ Labeling

Approximately 1 g muscle sample was ground into powder in liquid nitrogen. The powder was transferred into a tube with 700 μL extraction buffer (50 mM Tris-HCl (Trihydroxymethyl aminomethane hydrochloride) (pH = 7.5), 150 mM NaCl (Sodium chloride), 1 mM ethylenediaminetetraacetic acid (EDTA), 5 mM dithiothreitol (DTT), 1% Triton-X-100 and 7× protease inhibitor solution (Roche Applied Science, Indianapolis, IN, USA)) and mixed thoroughly. The mixed samples were then shaken for 30 min at 4 °C and centrifuged at 12,000× *g* for 10 min at 4 °C. The supernatant (protein extract) was transferred into a new marked tube. The protein concentration was determined using the bicinchoninic acid (BCA) protein assay (Shenggong, Shanghai, China). Two biological replicates were used in the case of each treatment type and storage time.

Next, a total of 100 μg protein of each sample was digested overnight at 37 °C with trypsin using a protein-to-trypsin ratio of 50:1 (Promega, Madison, WI, USA). iTRAQ labeling was performed using the iTRAQ^®^ Reagent-8-plex kit (AB SCIEX Inc., Redwood City, CA, USA) according to the manufacturer’s protocol. After trypsin digestion, the samples were centrifuged at 13,500× *g* for 12 min, re-dissolved in 50 μL 500 mM TEAB, marked with an isobaric tag, kept at a room temperature for 2 h and freeze-dried in vacuum. After lambing, the samples were collected for further analysis. All the labeled samples were mixed and preserved for further analysis.

#### 2.8.2. LC-MS/MS Analysis and Protein Identification

Each peptide mixture was dissolved into 100 μL buffer A (20 mM ammonium formate, pH 10.0) and separated by high-pH reversed-phase HPLC using the Ultimate 3000 system (Thermo Fisher Scientific, MA, USA) connected to an XBridge C18 column (4.6 mm ID, 250 mm length, 5 μm particles, Waters Corporation, Milford, MA, USA). The process was conducted using a linear gradient from 5% to 45% buffer B (80% acetonitrile, 20 mM ammonium formate, pH 10.0) in 40 min at a flow rate of 1 mL·min^−1^. The fractions were collected every 100 s and were eventually combined into 12 fractions based on collection time, followed by vacuum drying.

Separation of the fractions was conducted on an Easy-nLC 1000 system (Thermo Fisher Scientific, MA, USA) after dissolving the fractions in 0.1% formic acid. First, the fraction solution was loaded onto a trap column (Thermo Scientific Acclaim PepMap C18, 100 μm × 2 cm) at a flow rate of 10 μL·min^−1^. Next, the separation was performed using an analytical column (Acclaim PepMap C18, 75 μm × 15 cm) with a linear gradient of 2% to 40% buffer C (0.1% formic acid in 99.9% acetonitrile) for 70 min. An Orbitrap mass spectrometer (Orbitrap Fusion Tribrid^TM^, Thermo Fisher Scientific, MA, USA) was connected online to the Easy-nLC 1000 system with a nanospray ion source, which was used to analyze the data after full scans (*m*/*z* 350–1550) at a mass resolution of 120,000 as well as higher-energy collision dissociation (HCD) MS2 scans at a resolution of 30,000 with 30 s dynamic exclusion.

Raw data acquired from the Orbitrap mass spectrometer were converted to MGF files using the Mascot Distiller software. The Mascot search engine 2.3.2 (Matrix Science, London, UK, version 2.3.02) was used to identify the proteins. For protein quantitation, the protein species were required to contain at least two unique identifiable spectra. Quantitative protein rations were weighted and normalized by the median ration in Mascot. Protein expression in the CON and LP groups with a fold change larger than 1.2 or less than 0.83 and *P*-value < 0.05 was considered significantly different. The original data have been submitted to the ProteomeXchange database and assigned an internal ID of PXD011174.

#### 2.8.3. Bioinformatical Analysis

The Gene Ontology (GO) annotation and the Kyoto Encyclopedia of Genes and Genomes (KEGG) Pathway analysis of the DAPs were conducted using the KOBAS 2.0 (http://kobas.cbi.pku.edu.cn/) [37] and DAVID 6.7 (https://david.ncifcrf.gov/) [38] databases. The enriched GO terms were determined using a hypergeometric test, and those with a *P*-value < 0.05 were used to identify the significantly enriched GO terms and pathways.

#### 2.8.4. PRM-MS Analysis

The results of iTRAQ were verified by parallel reaction monitoring (PRM)-MS analysis. Eleven signature peptides for the target proteins were defined according to the iTRAQ data, and only unique peptide sequences were selected for PRM analysis. Sixty micrograms protein of each sample was extracted, reduced, alkylated and digested with trypsin following the procedure for iTRAQ analysis. The obtained peptide mixtures were introduced into the mass spectrometer via a C18 trap column (0.10 × 20 mm; 3 μm), and then through a C18 trap column (0.10 × 20 mm; 1.9 μm). A quadrupole mass filter-equipped bench-top Orbitrap mass spectrometer (Q-Exactive; Thermo Scientific) was used for MS measurements. Proteome Discoverer 1.4 (Thermo Fisher Scientific) was utilized to analyze the obtained raw data. Quantitative data processing and proteomic analyses were conducted using the Skyline 2.6 software.

### 2.9. Statistical Analysis

The MIXED procedure using SAS 8.0 (SAS Institute, Cary, NC, USA) with repeated measures was applied to analyze meat chemical composition and drip loss during post-mortem storage of various durations. Dietary treatment (T) effect (CON, LP200 and LP400), storage days (D) effect (1, 3, 5 and 7 d) and the interaction (T × D) were considered as fixed effects in a mixed model. Random effects were animals and random error. The variance of antioxidant indices which were affected by lycopene supplementation was analyzed using a one-way ANOVA in SAS 8.0. The Duncan Multiple Comparative Analysis was used to test significance among treatments (CON, LP200 and LP400), i.e., if lycopene indicated a significant effect on antioxidant parameters (*P* < 0.05). The replicate served as the experimental unit. Differences with a value of *P* < 0.05 were considered statistically significant. GraphPad Prism (version 7.0; GraphPad Software, Inc.) was used for figure plotting. 

## 3. Results

### 3.1. Meat Chemical Composition

The effect of dietary lycopene supplementation on the chemical composition of lamb meat during postmortem storage is shown in Table 2. No significant interaction effects between dietary lycopene supplementation and storage time were found for the measured meat chemical composition (*P*_Moisture_ = 0.4302, *P*_Crude protein_ = 0.0808, *P*_Ether extract_ = 0.8557, *P*_Crude ash_ = 0.0664). Moisture and ether extract contents were not affected by dietary lycopene supplementation on days 0 (*P*_Moisture_ = 0.3023, *P*_Ether extract_ = 0.1447) and 7 (*P*_Moisture_ = 0.9688, *P*_Ether extract_ = 0.2860). Compared with CON, crude protein content was higher (*P* = 0.0032) in the LP200 group on day 0. Ether extract content decreased (*P* = 0.0346) as the storage time increased in the CON group. Ash content in the CON group was lower (*P*_T_ = 0.0127) than that in the LP200 and LP400 groups on day 0 and decreased with storage time increase in the CON, LP200 and LP400 groups (*P*_CON_ < 0.0001, *P*_LP200_ < 0.0001 and *P*_LP400_ = 0.0004). 

### 3.2. Drip Loss During Storage Time

**Figure 1:**
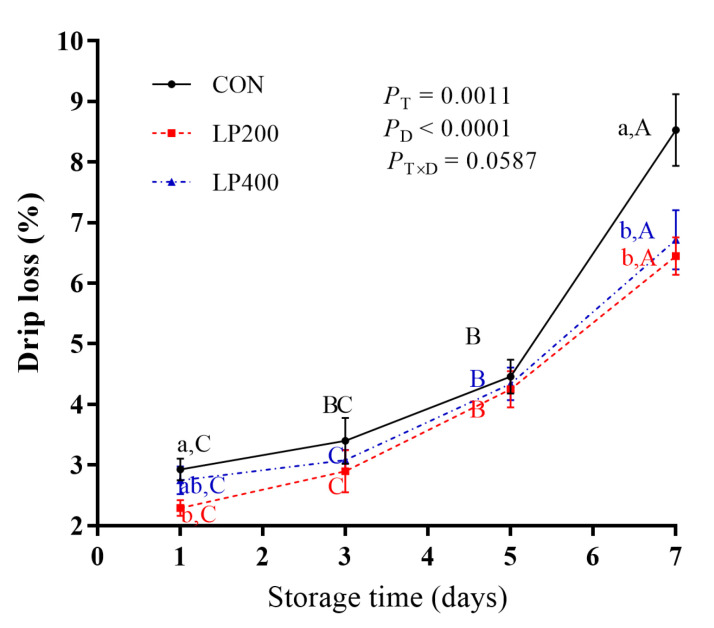
Effect of dietary lycopene supplementation on drip loss (on days 1, 3, 5 and 7) of longissimus thoracis (LT) muscle in lamb meat during storage (*n* = 10). *P*_T_, P-value of treatments; *P*_D_, P-value of storage days; *P*_T×D_, P-value of the interaction between treatments and storage days. Different capital letters indicate significant differences (*P* < 0.05) among storage time within treatment, and lowercase letters denote significant differences (*P* < 0.05) among treatments within time. The error bars represent standard error. CON, control group; LP200 and LP400 mean 200 or 400 mg·kg^−1^ lycopene powder supplementation in the control diet, respectively.

Drip loss increased significantly with storage days (*P* < 0.0001), but decreased with lycopene supplementation (*P* = 0.0011), reaching its highest value on day 7 (Figure 1). No significant interaction effect (*P* = 0.0587) was found between lycopene supplementation and storage days. Regardless of lycopene supplementation, drip loss increased slowly from day 1 to day 5, showing a sharp increase (*P* < 0.0001) from day 5 to day 7. Compared with CON, drip loss was lower (*P* = 0.0496) in LP200 on day 1 but was not different (*P* = 0.4817) in LP400. There was no significant difference between the CON and the lycopene-supplemented groups (LP200 and LP400) on day 3 (*P* = 0.5292) and day 5 (*P* = 0.8643). On day 7, drip loss of lamb meat following dietary lycopene addition (both LP200 and LP400) was lower (*P* = 0.0077) compared with CON, but there was no difference (*P* = 0.7018) between the LP200 and LP400 groups.

### 3.3. Antioxidant Capacity of Meat

**Figure 2:**
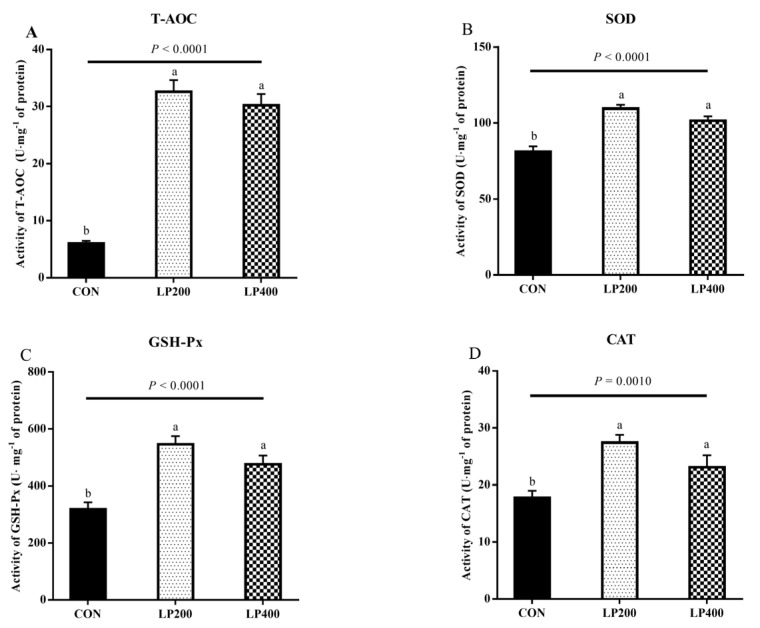
Effect of dietary lycopene supplementation on activities of antioxidant enzymes (on day 0)—total antioxidant capacity (T-AOC) (**A**), superoxide dismutase (SOD) (**B**), glutathione peroxidase (GSH-Px) (**C**) and catalase (CAT) (**D**) (*n* = 10). Different lowercase letters denote significant differences (*P* < 0.05) among treatments. The error bars represent standard error. CON, control group; LP200 and LP400 mean 200 or 400 mg·kg^−1^ lycopene powder supplementation in the control diet, respectively.

The activity of antioxidant enzymes in lamb meat with dietary lycopene addition is presented in Figure 2. The activities of T-AOC (Figure 2A), SOD (Figure 2B), GSH-Px (Figure 2C), and CAT (Figure 2D) increased (*P*_T-AOC_ < 0.0001, *P*_SOD_ < 0.0001, *P*_GSH-Px_ < 0.0001, *P*_CAT_ = 0.0010) significantly with dietary lycopene supplementation, while no difference (*P*_T-AOC_ = 0.3194, *P*_SOD_ = 0.0759, *P*_GSH-Px_ = 0.0836, *P*_CAT_ = 0.0693) was observed between the LP200 and LP400 groups.

### 3.4. Stability of Lipids and Proteins

**Figure 3:**
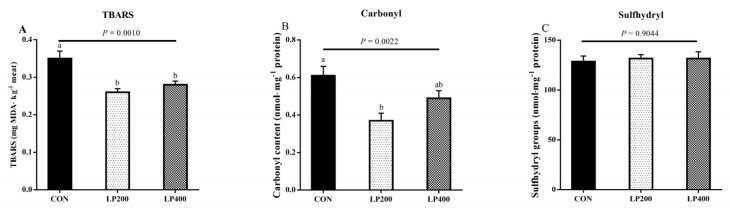
Thiobarbituric acid reactive substance (TBARS) (**A**), carbonyl (**B**) and sulfhydryl groups (**C**) content of muscle in response to lycopene added to the diet on day 0 (*n* = 10). Different lowercase letters indicate significant differences (*P* < 0.05) among treatments. The error bars represent standard error. NS, not significant. CON, control group; LP200 and LP400 mean 200 or 400 mg·kg^−1^ lycopene powder supplementation in the control diet, respectively.

TBARS content was the highest (*P* = 0.0010) in the CON treatment group and decreased upon 200 and 400 mg·kg^−1^ lycopene addition (Figure 3A); the different lycopene supplementation treatment groups had similar (*P* = 0.4720) TBARS contents. Compared with the CON group, the carbonyl content decreased (*P* = 0.0022) with 200 mg·kg^−1^ lycopene supplemented in the diet (Figure 3B). No significant differences (*P* = 0.9044) were found in the sulfhydryl contents among the three treatments (Figure 3C).

### 3.5. Proteomic Profiles

iTRAQ was utilized to analyze the differences in the proteomic profiles of lamb meat samples following dietary lycopene supplementation. Based on the results, there were no obvious difference between the LP200 and LP400 groups in terms of drip loss and antioxidant enzyme activities; thus, we examined the protein profiles between CON and LP200 (LP) to elucidate the effect of lycopene on lamb meat during the storage period (days 0, 5 and 7).

#### 3.5.1. Identification and Quantification of Protein Species

A total of 446,086 spectra were produced by iTRAQ analysis (Figure S1A in Supplementary Materials). Among them, 61,386 spectra were matched with known peptides, while we identified 6967 unique peptides and 1032 proteins (Figure S1A). The majority (69.69%) of the identified proteins’ molecular weights were located in the 10–20 (102), 20–30 (131), 30–40 (134), 40–50 (120), 50–60 (99) and 60–70 kD (83) ranges (Figure S1B). In addition, coverage rates of more than 10% and 20% of the identified proteins took the percentages of 52.33% and 31.59%, respectively (Figure S1C). The distribution of the peptides defining each protein number is shown in Figure S1D; we found that more than 66.67% of the proteins included at least two peptides.

The number of differentially abundant proteins (DAPs) on days 0, 5 and 7 is shown in Figure S2A. On day 0, 343 DAPs between LP-D0 and CON-D0 were identified, of which 178 were upregulated and 165 downregulated. Compared with CON-D5, 315 DAPs (196 up- but 119 downregulated) were found in LP-D5. On day 7, 214 DAPs were detected between LP-D7 and CON-D7, including 110 up- but 104 downregulated. A cluster heat map was plotted against the protein abundance of DAPs to visualize the variation in protein profiles (Figure S2B), which indicated that there was a marked alteration in the protein profiles in response to lycopene supplementation and storage time.

#### 3.5.2. Bioinformatical Analysis of DAPs

**Figure 4:**
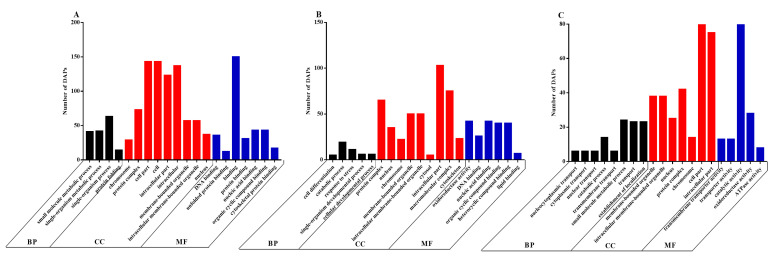
The 20 most enriched terms of differentially abundant proteins (DAPs) by Gene Ontology (GO) analysis between the CON and LP groups on days 0 (**A**), 5 (**B**) and 7 (**C**). BP, biological process; CC, cellular component; MF, molecular function. DAPs: differentially abundant proteins.

Functional analysis of the DAPs was performed based on the GO database. We found that the DAPs belonged primarily to the biological process (BP), cellular component (CC) and molecular function (MF) classes of proteins. The top 20 enriched GO terms between the CON and LP groups on days 0 (Figure 4A), 5 (Figure 4B) and 7 (Figure 4C) were selected to explore the functional changes of DAPs. The results indicated that most enriched DAPs belonged to the CC class. On days 0, 5 and 7, six identical categories (protein complex, intracellular part, membrane-bound organelle, intracellular membrane-bound organelle, nucleus and chromosome) in the CC class were found when comparing LP and CON groups. Moreover, on day 0, the most prominent BP categories included the small molecule metabolic process, the single-organism metabolic process and the single-organism process; the cell part, cell and intracellular were enriched in the CC class; and the most enriched terms in MF were binding, DNA binding, nucleic acid binding, organic cyclic compound binding and cytoskeletal protein binding. On day 5, few DAPs were enriched in the BP categories; the prominent terms in MF were oxidoreductase activity, nucleic acid binding, organic cyclic compound binding and heterocyclic compound binding. On day 7, few DAPs were involved in the BP categories; the cell part term also appeared in the CC category; the top terms in MF were catalytic activity and oxidoreductase activity.

**Figure 5:**
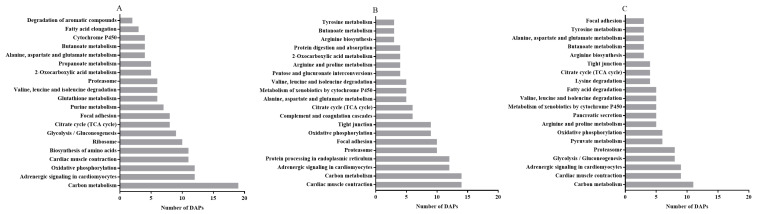
The 20 most enriched pathways of DAPs by Kyoto Encyclopedia of Genes and Genomes (KEGG) analysis between the CON and LP groups on days 0 (**A**), 5 (**B**) and 7 (**C**), arranged according to the number of DAPs. DAPs: differentially abundant proteins.

As the KEGG pathways analysis between the LP-D0 and CON-D0 groups revealed, on day 0, the predominantly enriched pathways were carbon metabolism, adrenergic signaling in cardiomyocytes, oxidative phosphorylation, cardiac muscle contraction, glycolysis/gluconeogenesis, citrate cycle (TCA cycle), focal adhesion and glutathione metabolism (Figure 5A) with lycopene supplementation. In addition, cardiac muscle contraction, carbon metabolism, adrenergic signaling in cardiomyocytes, protein processing in endoplasmic reticulum, proteasome, focal adhesion and oxidative phosphorylation were among the top KEGG pathways between the LP-D5 and CON-D5 groups after storage for 5 days (Figure 5B). With the exception of glycolysis/gluconeogenesis, the top five pathways between LP-D7 and CON-D7 were the same as those on day 5 (Figure 5C). When comparing the pathways among the three storage times, we found 10 enriched pathways that were the same on days 0, 5 and 7, as shown in Figure 5.

Based on the GO and KEGG results, we selected 10 candidate DAPs (oxidase: ubiquinone oxidoreductase core subunit S7 (NDUFS7); metabolic enzymes: succinate-CoA ligase, ADP-forming, subunit beta, mitochondrial (SUCLA2), succinate-CoA ligase, GDP-forming, subunit beta, mitochondrial (SUCLG2), proteasome 26S subunit, ATPase 2 (PSMC2) and proteasome subunit alpha type (PSMA3); calcium channel: calcium voltage-gated channel auxiliary subunit beta 1 (CACNB1); protein structure: Tropomyosin 3 (TPM3), troponin C1, slow skeletal and cardiac type (TNNC1), Myosin light chain (MYL)2 and MYL4) from days 0, 5 and 7 for further analysis to elucidate the underlying mechanism of lycopene supplementation on drip loss.

#### 3.5.3. Validation of DAPs by PRM

**Figure 6:**
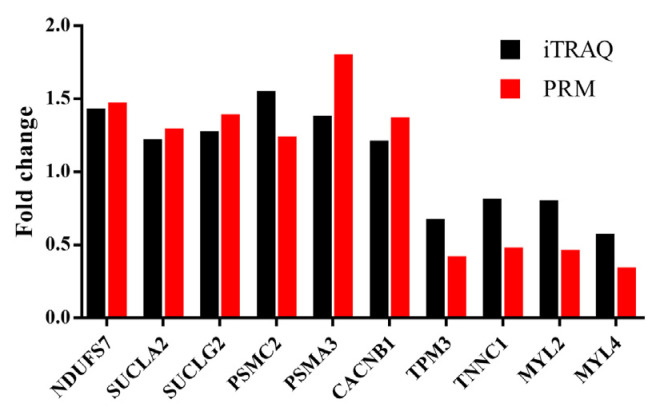
Verification of iTRAQ DAPs by using PRM analysis. NDUFS7, NADH: ubiquinone oxidoreductase core subunit S7; SUCLA2, Succinate-CoA ligase, ADP-forming, subunit beta, mitochondrial; SUCLG2, Succinate-CoA ligase, GDP-forming, subunit beta, mitochondrial; PSMC2, Proteasome 26S subunit, ATPase 2; PSMA3, Proteasome subunit alpha type; CACNB1, Calcium voltage-gated channel auxiliary subunit beta 1; TPM3, Tropomyosin 3; TNNC1, Troponin C1, slow skeletal and cardiac type; MYL2 and MYL4, Myosin light chain 2 and 4. iTRAQ: isobaric tags for relative and absolute quantitation; PRM: parallel reaction monitoring.

The PRM assay was used to validate the reliability of the iTRAQ analysis. We selected six upregulated DAPs (NDUFS7, SUCLA2, SUCLG2, PSMC2, PSMA3 and CACNB1) and four downregulated DAPs (TPM3, TNNC1, MYL2 and MYL4) for the assay. The expression level of the upregulated proteins was higher in the LP group than that in the CON group, and the downregulated proteins showed lower expression in the LP group when compared with that in the CON group. A significant correlation was found in the fold changes of these identified proteins between the LP and CON group (Pearson correlation coefficient r = 0.919; *P* < 10^−3^) (Figure 6).

## 4. Discussion

According to a recent report, the moisture, crude protein and crude ash contents of high-grade mutton are in the 70.80–80.25%, 18.50–23.39% and 0.79–1.18% ranges, respectively [39]. The values of these components for the meat used in the present study fell within those ranges, indicating its high quality. The protein content increased in the meat on day 0 when 200 mg·kg^−1^ lycopene was added to the diet, which may be due to the influence of dietary lycopene, which could promote protein deposition and muscular development by increasing the feed intake [19] and growth performance [28]. The result of crude fat decreasing to some extent was similar with results we reported previously, where dietary lycopene supplementation could reduce the fat content in Bamei lambs [19]. Its effect may be due to its ability to inhibit lipid synthesis by reducing cholesterol ester and triacylglycerol synthesis, as described in human macrophages [40]. Hayes et al. (2013) observed that ash content decreased as the tomato paste level increased in pork luncheon rolls during refrigerated storage [41], and our results are consistent with these data. However, no significant differences were found for moisture, protein, ether extract and ash content after 7 days of storage, which suggested dietary lycopene addition confers no obvious improvement to meat chemical composition from the aspect of long-term preservation.

Our current results indicate that drip loss increased with storage time, which is in accordance with findings reported previously [42]. Protein and lipid oxidation may also be responsible for the process due to the damage or transformation it causes in the structure of proteins and lipids [43,44]. The meat of lambs that received a lycopene-supplemented diet showed lower drip loss during the storage period, implying that lycopene addition during the fattening period could improve the WHC of meat during storage. Previous studies also reported that dietary supplementation of antioxidants could decrease drip loss of muscle in pigs [45,46], lambs [47] and in broilers [48].

Antioxidant enzymes play a critical role in the antioxidant defense system of organisms by protecting cells from oxidative damage caused by exogenous substances. SOD and GSH-Px are part of the first line of defense against antioxidants, in which SOD removes superoxide anion radicals by reacting directly with free radicals, while GSH-Px as an antioxidant enzyme catalyzes the decomposition of hydrogen peroxide [49]. Therefore, the increased activity of antioxidant enzymes in the meat of lambs that received a lycopene-supplemented diet indicates that lycopene may contribute to postmortem meat stability. However, no difference in antioxidant enzymes activity was found between the 200 and 400 mg·kg^−1^ lycopene supplementation groups; the underlying mechanism still needs further research.

TBARS and carbonyl are widely used for evaluating the extent of lipid and protein oxidation in muscle, respectively [50]. The TBARS and carbonyl contents of muscle decreased significantly in response to lycopene supplementation compared to the control group. TBARS level in muscle had an inverse relationship with the antioxidant level [13], and the degree of lipid oxidation could be reduced by adding antioxidants to the rations of the animals [51]. Lycopene has a strong inhibitory effect on lipid oxidation [52], and dietary lycopene supplementation could delay lipid oxidation in meat during storage (from day 1 to day 7 postmortem) [21]. Carbonyl content is in a direct relationship, whereas sulfhydryl groups are in a reverse relationship, with protein oxidation. Meat carbonyl levels decreased after dietary antioxidant supplementation [53], and they showed a positive correlation with drip loss [4]. Therefore, dietary lycopene supplementation improves the activity of antioxidant enzymes and enhances the lipid and protein stability of postmortem meat, which has a potential to decrease drip loss and may help to increase the WHC during storage.

Based on the biological function of the proteins from iTRAQ result, we carefully selected 10 DAPs and divided them into four categories (oxidases, metabolic enzymes, calcium channels and structural proteins) to analyze the key DAPs that lycopene works to decrease drip loss during storage. 

Oxidases: NDUFS7 is a subunit of one of the complexes that forms the mitochondrial respiratory chain and is part of the oxidative phosphorylation system [54]. The annotation of this protein can be found in relation to functions such as oxidoreductase activity, catalytic activity and binding. Oxidoreductase reactions are carried out by Respiratory Complex I (also known as NADH: ubiquinone oxidoreductase), located in the inner membrane of the mitochondria, and NDUFS7 as a subunit of NADH is responsible for the electron transfer to ubiquinone [55]. Therefore, NDUFS7 is essential for the assembly of Complex I, which is involved in oxidoreductase reactions. We found that on day 0 and day 5, the expression of NDUFS7 was higher in the meat of animals that received dietary lycopene supplementation compared to that in the control group. This might means that the electron clearance ability of the muscle enhanced and the oxidation reaction was inhibited. Thus, we consider that lycopene may protect muscles from oxidation damage through this mechanism to reduce drip loss. However, by day 7, NDUFS7 expression dropped to control levels, suggesting that lycopene may regulate drip loss by NDUFS7 but its effect does not last until day 7 of storage.

Metabolic enzymes: SUCLA2 and SUCLG2, which were enriched in the binding term of the GO analysis and are known to be involved in carbon metabolism and the citrate cycle (TCA cycle) pathway, showed higher expression following lycopene supplementation. SUCLA2 is the beta-subunit of succinyl-CoA ligase and shows specificity for the GDP subunit [56]. Lower expression of SUCLA2 and SUCLG2 in the case of the control animals with no lycopene supplementation results in the formation of fewer ATP molecules [57]. Low ATP levels lead to limited electron transfer activity, an increase in anaerobic respiration and, thus, the production of more lactic acid, which eventually decreases the pH of the meat. A lower pH value can cause some proteins to reach their isoelectric point (pI), which results in weakened WHC and a much higher drip loss [58]. The expression of PSMC2 and PSMA3 was higher with dietary lycopene supplementation, especially on days 5 and 7; these proteins are part of the proteasome pathway. According to a previous report, the proteasome promotes the degradation of most proteins in vitro [59], while the results of more recent studies indicated that it was able to improve the WHC of myofibrils via proteolytic degradation of myofibrillar proteins [45,60]. We surmise that dietary lycopene supplementation perhaps not only improved antioxidant capacity by increasing the amount of metabolic enzymes involved in electron transfer, but also contributed to structural protein degradation, which caused improvement in the solubility of protein and increased the space for water storage in the meat during storage.

Calcium channels: CACNB1 belongs to the calcium channel beta subunit family and is involved in the cardiac muscle contraction pathway. It plays an important role in the proper functioning of the calcium channel by modulating G protein inhibition, increasing peak calcium current and regulating calpain activity [61]. Calpain activity has a strong negative correlation with drip loss [62,63], which may be attributable to the fact that calpain can degrade the myofibril protein network, which results in a structural change that subsequently allows increased swelling of the myofibrils as water flows from the extracellular to the intracellular space [60]. At the same time, calpain activity is also affected by oxidative reactions and is inhibited by oxidants [64,65]. Therefore, dietary lycopene supplementation may decrease drip loss by increasing the abundance of CACNB1 and also the antioxidant capacity to maintain calpain activity during storage.

Structural proteins: Myosin is the most abundant protein in the body and makes up about one-third of the total muscle proteins [66]. Through its ATPase activity, it can hydrolyze ATP to provide energy for muscle contraction [11]. The myosin content of muscles was known to have a direct relationship with drip loss [67]. In the present study, the expression of TPM3, TNNC1, MYL2 and MYL4 decreased following lycopene supplementation and the progress of storage time; these known structural proteins were enriched in the muscle contraction pathway category. These results suggest that a decrease in the structural protein content reduced muscle contraction, and at the same time, it provided more space for water holding [68]. In addition, myosin as the major protein related to water holding in muscle slowed down the process of reaching the pI, which keeps the thick and thin myofilaments at a relatively stable distance [69]. Meanwhile, in the CON group, because of a drop in pH during storage [21], the distance between thick and thin myofilaments decreased and sarcomere length shortened. Muscle contraction was then enhanced because a lower pH value allowed myosin to reach the pI faster, which eventually led to water being squeezed out of the cell during muscle contraction. Therefore, drip loss was decreased during storage, perhaps by reducing the amount and contraction of structural proteins to provide more space for water holding.

Though dietary lycopene supplementation may help to improve meat quality and antioxidant capacity, the process of lycopene metabolism, especially in ruminant animals, is still unclear, and the potential mechanism between candidate DAPs and drip loss during storage deserve more attention and further study.

## 5. Conclusions

In the present study, dietary lycopene supplementation decreased drip loss of lamb meat during storage and increased the activity of antioxidant enzymes and the stability of lipids and proteins in the meat after slaughtering. Based on the iTRAQ data of meat during storage, we identified 10 proteins (NDUFS7, SUCLA2, SUCLG2, PSMC2, PSMA3, CACNB1, TPM3, TNNC1, MYL2 and MYL4) that are involved in the oxidative phosphorylation, carbon metabolism, citrate cycle (TCA cycle), proteasome and cardiac muscle contraction pathways. These results suggest that dietary lycopene supplementation can increase the antioxidant capacity of postmortem lamb meat and may reduce drip loss during storage by regulating the expression of oxidases, metabolic enzymes, calcium channels and structural proteins. Based on the findings of the current study, the optimum lycopene addition level is 200 mg/kg DM in the diet.

## Figures and Tables

**Table 1:** Ingredients and chemical composition of the basic diet.

Ingredient (g·kg^−1^ Fed Basis)	Content	Chemical Composition (g·kg^−1^ DM Basis)	Content
Corn silage	220	Dry matter	890
Peanut vine	280	Crude protein	150
Corn	290	Ether extract	23.8
Soybean meal	145	Neutral detergent fiber	272
Wheat bran	30	Acid detergent fiber	180
Salt	5	ME ^2^ (MJ·kg^−1^)	10.14
Sodium bicarbonate	5	Calcium	7.5
Premix ^1^	25	Phosphorus	3.6

^1^ The premix provided the following kg^−1^ diets: Vitamin A 5500 IU, Vitamin D 1750 IU; Fe 40 mg; Cu 7.5 mg; Mn 30 mg; Zn 45 mg; I 0.65 mg; Se 0.14 mg; Co 0.25 mg. ^2^ Metabolizable energy (ME) was a calculated value, while the others were measured values. DM: dry matter.

**Table 2:** Effect of dietary lycopene supplementation on the chemical composition of lamb meat on days 0 and 7 of storage (g 100 g^−1^ fresh meat).

Item	Days (D)	Treatments (T)			*P*-Value				
		CON	LP200	LP400	T	D			T × D
						CON	LP200	LP400	
Moisture	0	73.79 ± 0.86	75.00 ± 0.30	74.46 ± 0.33	0.3023	0.5093	0.1796	0.9680	0.4302
	7	74.41 ± 0.19	74.38 ± 0.31	74.48 ± 0.40	0.9688				
Crude protein	0	19.74 ± 0.14 ^b^	21.56 ± 0.41 ^a^	20.63 ± 0.36 ^a,b^	0.0032	0.2369	0.0871	0.6231	0.0808
	7	20.20 ± 0.38	20.44 ± 0.35	20.39 ± 0.23	0.8622				
Ether extract	0	3.44 ± 0.21 ^A^	2.92 ± 0.14	3.09 ± 0.19	0.1447	0.0346	0.0705	0.0880	0.8557
	7	2.85 ± 0.19 ^B^	2.53 ± 0.12	2.66 ± 0.10	0.2860				
Ash	0	1.77 ± 0.08 ^a,A^	1.49 ± 0.05 ^b,A^	1.47 ± 0.07 ^b,A^	0.0127	0.0000	0.0000	0.0004	0.0664
	7	1.12 ± 0.02 ^B^	1.08 ± 0.02 ^B^	1.08 ± 0.02 ^B^	0.1998				

Different lowercase letters in the same row show significant differences in dietary lycopene supplementation effects (*P* < 0.05); different capital letters in the same row indicate significant differences between days 0 and 7. CON, control group; LP200 and LP400 mean 200 or 400 mg·kg^−1^ lycopene supplementation in the control diet, respectively; T, treatments; D, storage days; T × D, interaction effects between treatments and storage days; results are shown as mean ± SEM. *N* = 10 for each treatment on days 0 and 7.

## Data Availability

The original data of proteomics have been submitted to the ProteomeXchange database and assigned an internal ID of PXD011174.

## Supplementary Materials

The following are available online at https://www.mdpi.com/2076-3921/10/2/198/s1, Figure S1: Overview of quantitative proteomics; Figure S2: Differentially abundant proteins number and Heatmap between the CON and LP groups on days 0, 5 and 7.
