# Supplementary material for: The Effect of Dietary Lycopene Supplementation on Drip Loss during Storage of Lamb Meat by iTRAQ Analysis
Source: Antioxidants (Basel). 2021 Jan 29;10(2):198. doi: 10.3390/antiox10020198 (PMC7911479; doi:10.3390/antiox10020198)
Supplement: Supplementary file 1 [file antioxidants-10-00198-s001.pdf]

Supplementary figures

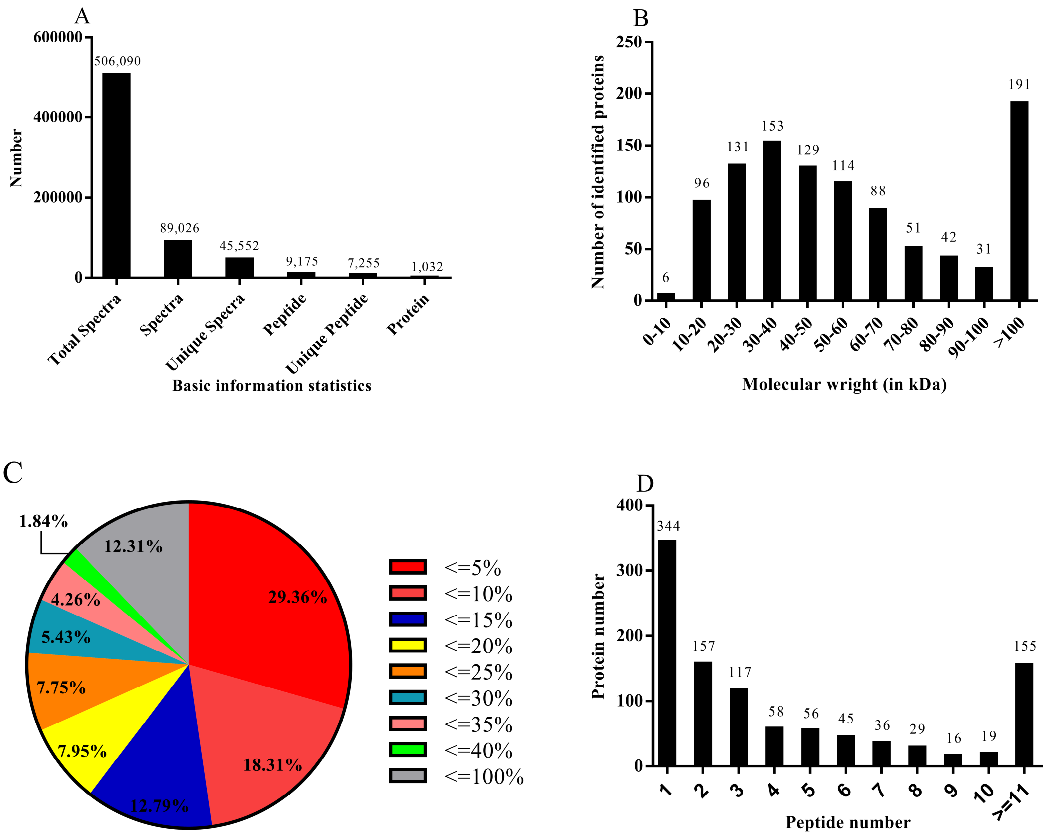

Figure S1. Overview of quantitative proteomics. (**A**) Basic information statistics of protein identification, (**B**) distribution of the identified proteins according to molecular weight (in kD), (**C**) coverage (i.e., percentage of protein sequences covered by identified peptides), and (**D**) distribution of the identified peptides according to their numbers in proteins.

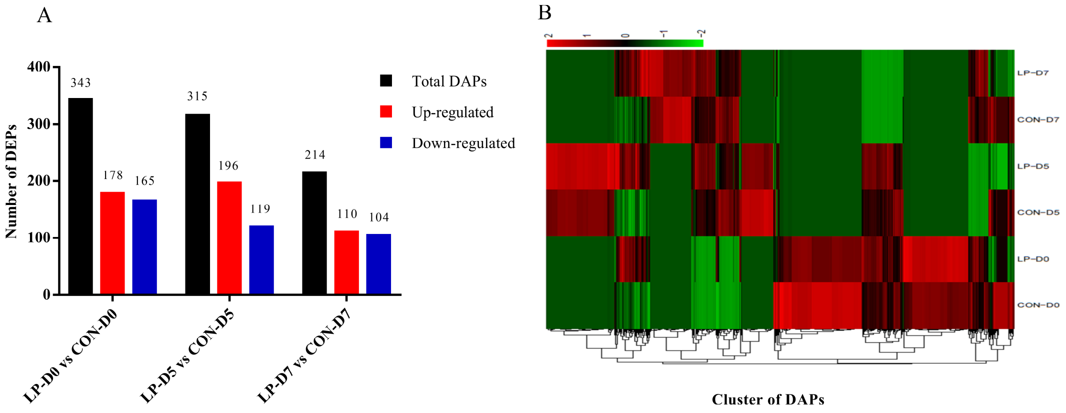

Figure S2. Differentially abundant proteins number and Heatmap between the CON and LP groups on days 0, 5 and 7. (A) Number of differentially expressed proteins on days 0, 5 and 7 between the control and lycopene supplementation group. LP-D0, LP-D5 and LP-D7 means lycopene supplementation group on day 0, 5 and 7, respectively. CON-D0, CON-D5 and CON-D7 means control group on day 0, 5 and 7, respectively. (**B**) Heatmap based on cluster analysis of DAPs following different storage time between the CON and LP groups. DAPs: differentially abundant proteins.
